# Novel Aryl Hydrocarbon Receptor Agonist Suppresses Migration and Invasion of Breast Cancer Cells

**DOI:** 10.1371/journal.pone.0167650

**Published:** 2016-12-01

**Authors:** Hamza Hanieh, Omar Mohafez, Villianur Ibrahim Hairul-Islam, Abdullah Alzahrani, Mohammad Bani Ismail, Krishnaraj Thirugnanasambantham

**Affiliations:** 1 Biological Sciences Department, College of Science, King Faisal University, Hofuf Ahsaa, Saudi Arabia; 2 Biomedical Department, College of Clinical Pharmacy, Hofuf Ahsaa, Saudi Arabia; 3 Biochemistry Department, College of Pharmacy Al-Azhar University Assiut, Egypt; 4 Pondicherry Centre For Biological Sciences, Jawahar Nagar, Pondicherry, India; Nihon University School of Medicine, JAPAN

## Abstract

**Background:**

Despite the remarkable progress to fight against breast cancer, metastasis remains the dominant cause of treatment failure and recurrence. Therefore, control of invasiveness potential of breast cancer cells is crucial. Accumulating evidences suggest Aryl hydrocarbon receptor (Ahr), a helix-loop-helix transcription factor, as a promising target to control migration and invasion in breast cancer cells. Thus, an Ahr-based exploration was performed to identify a new Ahr agonist with inhibitory potentials on cancer cell motility.

**Methods:**

For prediction of potential interactions between Ahr and candidate molecules, bioinformatics analysis was carried out. The interaction of the selected ligand with Ahr and its effects on migration and invasion were examined *in vitro* using the MDA-MB-231 and T47D cell lines. The silencing RNAs were transfected into cells by electroporation. Expressions of microRNAs (miRNAs) and coding genes were quantified by real-time PCR, and the protein levels were detected by western blot.

**Results:**

The *in silico* and *in vitro* results identified Flavipin as a novel Ahr agonist. It induces formation of Ahr/Ahr nuclear translocator (Arnt) heterodimer to promote the expression of cytochrome P450 family 1 subfamily A member 1 (Cyp1a1). Migration and invasion of MDA-MB-231 and T47D cells were inhibited with Flavipin treatment in an Ahr-dependent fashion. Interestingly, Flavipin suppressed the pro-metastatic factor SRY-related HMG-box4 (Sox4) by inducing miR-212/132 cluster. Moreover, Flavipin inhibited growth and adhesion of both cell lines by suppressing gene expressions of B-cell lymphoma 2 (Bcl2) and integrinα4 (ITGA4).

**Conclusion:**

Taken together, the results introduce Flavipin as a novel Ahr agonist, and provide first evidences on its inhibitory effects on cancer cell motility, suggesting Flavipin as a candidate to control cell invasiveness in breast cancer patients.

## Introduction

Breast cancer is a dominant cause of cancer-related death among cancer patients worldwide [1]. Despite the significant progress to fight against malignancy, the invasiveness and metastasis of breast cancer contribute to the high mortality rate [2]. In addition, the toxicity of the available drugs is a persistent problem in effective chemotherapies [3]. Therefore, development of drugs that show effective anticancer properties with less toxic effects is a priority [4, 5]. Recent decades have witnessed constant emergence of candidate molecules that show effective inhibition of cell motility in breast cancer [6–8]. However, there is a need for safe molecules of natural origin.

The Aryl hydrocarbon receptor (Ahr) is a heterodimeric protein belongs to the helix-loop-helix/Per-Arnt-Sim (bHLH/PAS) family of transcription factors. It is activated by wide range of natural and synthetic molecules. Upon ligation, Ahr heterodimerizes with the Ahr nuclear translocator (Arnt) to modulate cell functions through downstream genes such as cytochrome P450 (Cyp) [9, 10]. Recent paradigm of promising anti-breast cancer candidates has revealed that Ahr mediates their therapeutic potential. For example, (5S,7S)-7-methyl-3-(3-(trifluoromethyl)phenyl)-5,6,7,8-tetrahydrocinnolin-5-ol (NK150460) suppresses growth of different estrogen receptor (ER)-negative and ER-positive breast cancer cell lines via the molecular cascade of Ahr/Arnt [11]. The Ahr mediates the inhibitory effects of 2-(4-hydroxy-3-methoxyphenyl)-benzothiazole (YL-109) on growth and invasion of MDA-MB-231 through upregulation of Hsp70-interacting protein (CHIP) [12]. Furthermore, the icaritin derivative of prenylflavone inhibits cell growth of MCF-7 cells by destabilization of ERα in an Ahr-dependent fashion [13]. Due to the therapeutic potential role of Ahr against breast cancer, research efforts have been directed towards identification of new Ahr ligands.

Importantly, Ahr has emerged as a potential therapeutic strategy to control motility of breast cancer cells including migration, invasiveness and metastasis. For example, omeprazole-activated Ahr based down-regulation of matrix metalloproteinase-9 (MMP-9) and C-X-C chemokine receptor 4 (CXCR4) suppresses invasion and metastasis of MDA-MB-231 cells [14]. The 3,3′-diindolylmethane (DIM) suppresses invasion of MCF-7 and T47D by inhibition of phosphorylation of type III epidermal growth factor receptor mutation (EGFRvIII) [15]. Previously, we found that DIM and the synthetic toxin 3,7,8-tetrachlorodibenzo-p-dioxin (TCDD) suppress migration, invasion and metastasis of MDA-MB-231 and T47D in an Ahr-dependent fashion [16]. These suppressive effects of Ahr agonists on cell motility were attributed mainly to the microRNA (miR)-212/132 cluster that targets the pro-metastatic SRY-related HMG-box4 (Sox4). In lines, TCDD induces Sox4-targeting miR-335 to inhibit the motility of MDA-MB-231 [17].

Flavonoids are a wide range of natural compounds that have been intensively studied for their anticancer properties [18, 19]. Interestingly, some flavonoid compounds exert their effects through activation of Ahr [20, 21]. Unlike most of the known flavonoids, few studies have been performed to study the biological significance of Flavipin [22, 23]. In addition, no studies have been performed to investigate the anticancer potential of this phenolic compound.

Herein, we applied an *in silico* and *in vitro* approach to examine a potential interaction between Flavipin and Ahr in relation to breast cancer. For the first time, we introduced Flavipin as a novel Ahr agonist, and we provided evidences for its inhibitory activity on migration and invasion of ER-negative and ER-positive breast cancer cell lines. In addition, we suggested a precise molecular explanation of the reported biological effects.

## Materials and Methods

### *In silico* analysis

Potential interactions between Ahr and candidate ligands including Flavipin, Epicorazines A, Epicorazines B and Lonchocarpol A were examined by means of docking analysis using Autodock tools (ADT) v1.5.4 and Autodock v4.2 program (http://www.scripps.edu/mb/olson/doc/autodock). The 3D chemical structure of Ahr PAS-A (PDB ID: 4M4X) was retrieved from the Protein Data Bank (http://www.pdb.org), and that of Flavipin (CID_3083587), Epicorazines A (CID_ 57383998), Epicorazines B (CID_ 73891006) and Lonchocarpol A (CID_124035) were retrieved from Pubchem compound database (http://www.ncbi.nlm.nih.gov/pccompound). The active sites of the target protein were identified using Q-site Finder. Ligands docked to the receptor were reflected as a rigid body, and receptor was considered as flexible factor. The results were evaluated and sorted based on predicted binding energy.

The Ahr molecule is composed of PAS-A and PAS-B domains. Due to unavailability of the 3D structure of human Ahr PAS-B, a model was predicted and docked against Flavipin. Peptide sequence of human Ahr PAS-B (NP_001612.1) was retrieved from NCBI (https://www.ncbi.nlm.nih.gov/protein/). The 3D structure of Ahr PAS-B was predicted based on human Arnt PAS-B (PDB ID: 2B02) obtained from Protein Data Bank as described earlier [24]. To predict the PAS-B 3D structure we used PS2 software (http://www.ps2.life.nctu.edu.tw/). The developed 3D model was validated for stereochemical quality using SAVES software; https://services.mbi.ucla.edu/SAVES/, and for superimposition with human Arnt PAS-B using SALIGN software (http://salilab.org/salign). Finally the predicted 3D structure was visualized by UCSF Chimera (http://www.cgl.ucsf.edu/chimera/).

### Cell culture

The human ER-negative MDA-MB-231 and ER-positive T47D breast cancer cell lines were purchased from the American Type Culture Collection (ATCC; VA, USA). Dulbecco’s modified Eagle’s medium (DMEM)/Ham’s F12 (F12) containing fetal bovine serum (FBS) (Sigma-Aldrich, MO, USA) and 1% antibiotic antimycotic agent (Gibco, MD, USA) was used for maintaining the cells (complete medium). Cells were incubated in humidified incubator at 37°C and 5% CO_2_.

### Cell proliferation and adhesion

The MDA-MB-231 and T47D cells were suspended at 2.0×10^4^ in a medium containing 2.5% charcoal-stripped FBS and seeded in 96-well plate, and then treated for 48 h with DMSO or Flavipin. The T47D medium contained 10 nmol/L β-estradiol (E2; Santa Cruz Biotechnology, CA, USA). Cell proliferation was quantified calorimetrically at 540 nm using Cell-counting Kit-8 (CCK-8; Dojindo, MD, USA) following manufacturer’s instructions. For cell adhesion, 5×10^5^ cells were seeded in 6-well plate and incubated overnight in complete medium to attach, then washed with PBS to remove the non-adherent cells. Cells were then treated with DMSO or Flavipin in the charcoal-stripped medium for 48 h, and washed twice with PBS before microscopic examination.

### Cell migration and invasion

For migration assay, the cells were seeded in 6-well plate in a complete medium and allowed to attach overnight. The media were then changed to DMEM/F12 with 2.5% charcoal-stripped FBS containing DMSO or Flavipin for 24 h. Around 70% cell confluency, a line was made at the central axis of the wells using pipette tip, the wells were then gently washed and treated again with either DMSO- or Flavipin-containing medium for another 24 h. The T47D medium was supplemented with E2. The invasion assay was performed using a matrigel-coated Boyden chamber (8μm PET, Corning, NY, USA) as previously described [16]. Briefly, 2.0×10^4^ cells were suspended in 200 μL serum-free medium containing either DMSO or Flavipin and seeded in the trans-wells, and 750 μL complete medium was placed in the lower wells. After 24 h incubation, the cells on the trans-wells were fixed with 10% formaldehyde and permeabilized with methanol then stained with Giemsa. Cells with spread-out shape on the lower side of the trans-well were counted in 4 microscopic fields.

### Quantitative real-time PCR

Total RNA was extracted using RNeasy extraction kit (Qiagen, CA, USA) and cDNA was prepared using reverse transcriptase. The DNA amplification was carried out in ViiA7 real-time PCR system with the conditions of 50°C for 2 min and 95°C for 10 min, then 40 cycles of 95°C for 15 s and 60°C for 1 min. The TaqMan® gene expression assays of Cyp1a1 (Hs01054797_g1), B-cell lymphoma 2 (Bcl2; Hs01048932_g1), Sox4 (Hs00268388_s1), integrinα4 (ITGA4; Hs00168433_m1), and TaqMan® microRNA assay of hsa-miR-132 (ID: 00457) and hsa-miR-212 (ID: 00515) were used. The GAPDH (Hs02758991_g1) was used as an internal control for protein-coding genes, and RNU6B (ID: 001093) was used for miRNAs. Relative expressions were calculated using the ΔΔCt method. Machines, kits and reagents for cDNA synthesis and real-time PCR were purchased from Applied Biosystems, NY, USA.

### Western blot

Cells were lysed using RIPA lysis buffer system (Santa Cruz Biotechnology), and then fractionated using SDS-page. The proteins of Cyp1a1, Arnt, Bcl2, Sox4, ITGA4 and β-actin were detected using their specific rabbit polyclonal antibodies (Santa Cruz Biotechnology). Band intensities were quantified using ImageJ v.1.48 software http://imagej.nih.gov/ij/download.html [25].

### Cell transfection

The silencing RNA for Ahr (siAhr) and non-specific control (siNS) (Ambion, TX, USA) were transfected at 75 nmol/L, and pWPXL-Sox4 plasmid (36984; Addgene, MA, USA) or empty plasmid were transfected at 200 μg. The oligonucleotides were transfected into cell lines using 4D-nucleofector device and cell-specific transfection kits (Lonza, MD, USA). Transfected cells were allowed to recover for 6 h, and then the transfection medium was changed to a complete DMEM/F12 medium with DMSO or Flavipin. Transfection efficiency was confirmed by real-time PCR and western blot assays.

### Statistical analysis

Data are shown as mean ± SD from representative experiment studied in triplicates out of three independent experiments produced similar results. The analysis of variance (ANOVA) test was used to analyze the statistical significance. P<0.05 was considered significant.

## Results

### Flavipin interacts with Ahr *in silico*

**Fig 1:**
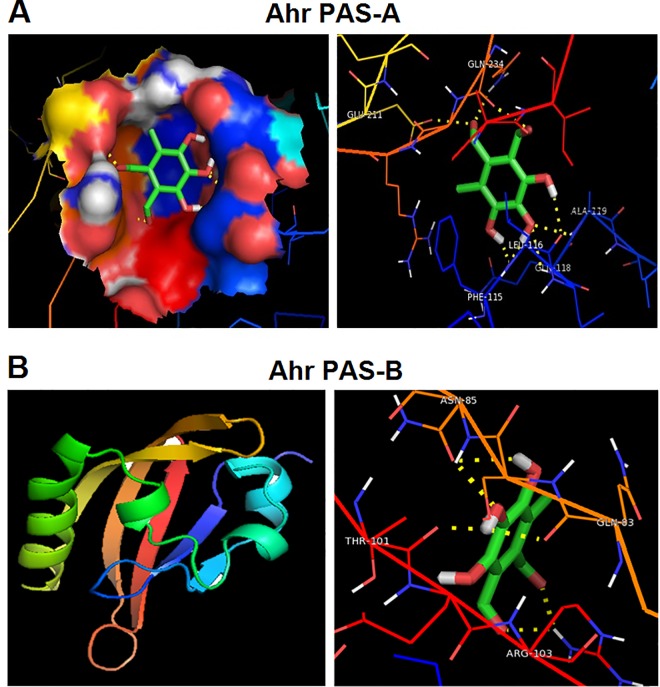
The *in silico* docking of Flavipin binding potential to human Ahr Pas-A and Pas-B Ligand domain. (A) Flavipin binds to Ahr PAS-A at Phe115, Leu116 and Ala119. (B) The Flavipin interacted with the four active residues of Ahr PAS-B at Gln83, Asn85, Thr101 and Arg103. The yellow-dotted lines indicates the hydrogen bonds.

Four natural compounds from flavonoids family, which have never been investigated for their potential anticancer properties, were chosen to examine their potential interaction with Ahr. These include Flavipin, Lonchocarpol A, Epicorazines A and Epicorazines B. The preliminary analysis using docking approach revealed that Flavipin and Lonchocarpol A significantly interact with Ahr PAS-A with binding energy of -4.63 and -4.35 kcal/mol, respectively (Table 1 and S1 Table). The Flavipin interacted with the three active residues of Ahr at Phe115, Leu116 and Ala119 (Fig 1A), whereas Lonchocarpol A interacts with two residues at Thr264 and Ala119 (S1 Fig). The potential interaction between Flavipin and Ahr formed eight hydrogen bonds, whereas that of Lonchocarpol A formed two bonds. The higher numbers of hydrogen bonds and amino acids involved in Flavipin-Ahr interaction reflected higher binding efficiency, which prompted us to choose Flavipin for further investigations.

**Table 1:** Molecular docking of Flavipin with Ahr PAS-A and PAS-B domain.

Ligand	Receptor	Binding energy	Ligand efficiency	Intermole energy	Ligand atoms (ring)	Docked amino acid residue (bond length)
Flavipin	Ahr PAS-A	-4.63	-0.34	-5.56	• C-7’O	• GLU`211/OE2 (2.7 Å)
					• C-7’O	• GLN`234/2HE2 (2.8 Å)
					• C-8’O	• GLN`234/O (2.6 Å)
					• C-5’OH	• LEU`116/O (2.3 Å)
					• C-4’O	• ALA`119/HN (2.4 Å)
					• C-4’O	• GLN`118/HN (2.4 Å)
					• C-4’OH	• PHE`115/O (1.7 Å)
					• C-3’OH	• PHE`115/O (2.1 Å)
Flavipin	Ahr PAS-B	-4.31	-0.31	-5.31	• C-8’O	• ARG`103/1HH1 (2.2 Å)
					• C-7’O	• ARG`103/1HH1 (2.2 Å)
					• C-4’OH	• THR`101/O (1.8 Å)
					• C-4’OH	• GLN`83/O (2.2 Å)
					• C-4’H	• ASN`85/HN (2.3 Å)
					• C-3’OH	• ASN`85/OD1 (2.3 Å)

The PAS-B domain is vital for interaction of Ahr with its ligands. Therefore, the 3D structure of human Ahr PAS-B domain was predicted. The primary structure properties, amino acid composition, Ramachandran analysis and plot, and G-factor parameters are presented in S2–S4 Tables and S2 Fig. Docking analysis of the predicted 3D structure revealed that Flavipin interacts with Ahr PAS-B domain with binding energy of -4.31 kcal/mol (Table 1). The Flavipin interacted with the four active residues of Ahr at Gln83, Asn85, Thr101 and Arg103 (Fig 1B). The potential interaction between Flavipin and Ahr formed six hydrogen bonds.

### Flavipin activates Ahr to recruit Arnt and induce Cyp1a1

**Fig 2:**
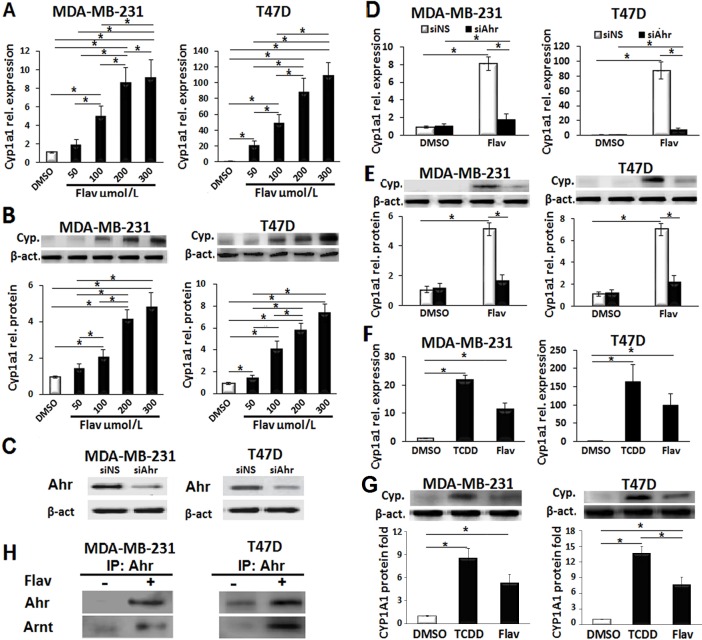
Flavipin induces Cyp1a1 gene expression in an Ahr-dependent fashion activity in breast cancer cell lines. (A, B) The mRNA and protein levels of Cyp1a1 in MDA-MB-231 and T47D cells were elevated after treatment with Flavipin for 48 h. Quantification was performed by real-time PCR and western blot. (C) Confirmation of siAhr efficiency in breast cancer cells. (D, E) The siAhr inhibited the increase in Cyp1a1 mRNA and protein induced by Flavipin (200 μmol/L). (F, G) Comparison of Cyp1a1 gene expression in response to TCDD or Flavipin in MDA-MB-231 and T47D cells. (H) Flavipin induced Ahr/Arnt complex. Immunoprecipitation (IP) using Ahr antibodies was performed and precipitated Ahr and Arnt were detected by western blot. Data are shown as mean ± SD from representative experiment studied in triplicates. *P<0.05.

When Ahr is ligated, it typically heterodimerizes with Arnt to promote the transcription of the P450 downstream genes. The Cyp1a1 is a member of the P450 family that is often used as an indicator of Ahr activation. Therefore, we first examined whether Flavipin induces gene expression of Cyp1a1 in breast cancer cells. The mRNA and protein quantifications presented in Fig 2A and 2B showed that Flavipin induced Cyp1a1 gene expression in MDA-MB-231 and T47D cells in a concentration-dependent fashion (50–300 μmol/L). Next, to confirm that Flavipin induced Cyp1a1 in an Ahr-dependent fashion, we depleted Ahr using siAhr. The efficiency of Ahr inhibition was confirmed by western blot as shown in Fig 2C. As depicted in Fig 2D and 2E, knockdown of Ahr drastically abolished the effect of Flavipin on Cyp1a1 gene expression.

The 2,3,7,8-tetrachlorodibenzo-p-dioxin (TCDD) is the most potent Ahr agonist that binds with high affinity. Therefore, we compared the effect of Flavipin and TCDD on Cyp1a1 gene expression. There were no significant differences in the mRNA levels of Cyp1a1 in both breast cancer cell lines (Fig 2F), but the protein levels of Cyp1a1 in Flavipin-treated cells were lower than TCDD (Fig 2F and 2G). Finally, to study whether Flavipin could recruit the Arnt subunit to form complex with Ahr, we performed immunoprecipitation (IP). The western blot analysis in Fig 2H confirmed formation of the Ahr/Arnt complex in response to Flavipin treatment. Taken together, the results suggest Flavipin as a novel Ahr agonist.

### Flavipin inhibits proliferation, adhesion, migration and invasion of breast cancer cells

**Fig 3:**
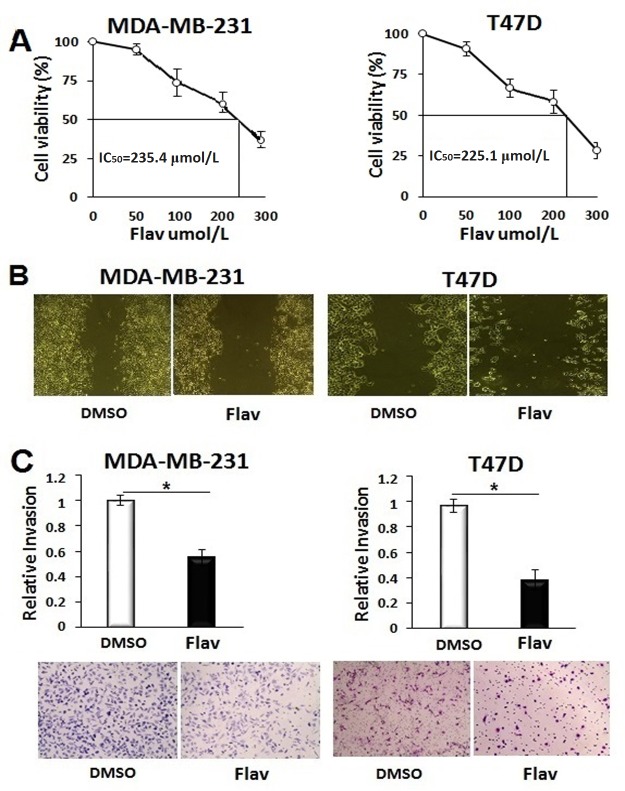
Flavipin inhibits cell migration and invasion of breast cancer cell lines. (A) The IC_50_ of Flavipin in MDA-MB-231 and T47D cells after 48 h treatment. Cell viability was quantified calorimetrically at 540 nm using CCK-8 kit. (B) Migration of MDA-MB-231 and expansion of T47D were inhibited 24 h after Flavipin treatment (200 μmol/L). (C) Cell invasion of MDA-MB-231 and T47D was suppressed 24 h after Flavipin treatment. Invasiveness of cancer cells were studied using Boyden chamber. The invaded cells were stained with Giemsa and counted in 4 different microscopic fields. Data are shown as mean ± SD from representative experiment studied in triplicates. *P<0.05.

Before investigating the biological significance of Flavipin, we determined the IC_50_ values. Quantification of cell proliferation of MDA-MB-231 and T47D cells 48 h after Flavipin treatment showed that the values of IC_50_ of Flavipin were 235.4 and 225.1 μmol/L, respectively (Fig 3A). Therefore, the Flavipin at a concentration of 200 μmol/L was used for the rest of the study. The Flavipin treatment exerted robust inhibitory effects on adhesion of T47D cells, and to a lesser extent, that of MDA-MB-231 cells (S3 Fig).

Migration and invasion of the breast cancer cells were studied to evaluate the effect of Flavipin on cell motility. Flavipin suppressed migration of MDA-MB-231 and proliferation-based expansion of T47D (Fig 3B). These inhibitory effects of Flavipin on proliferation and adhesion might have contributed to its suppressive effects on migration of the breast cancer cells. The Boyden chamber assay was used to study the effect of Flavipin on cell invasiveness capacity. The results showed that the number of invaded cells were reduced by 50% in MDA-MB-231 and 62% in T47D cells (Fig 3C).

### The inhibitory effects of Flavipin on breast cancer cells are Ahr-dependent

**Fig 4:**
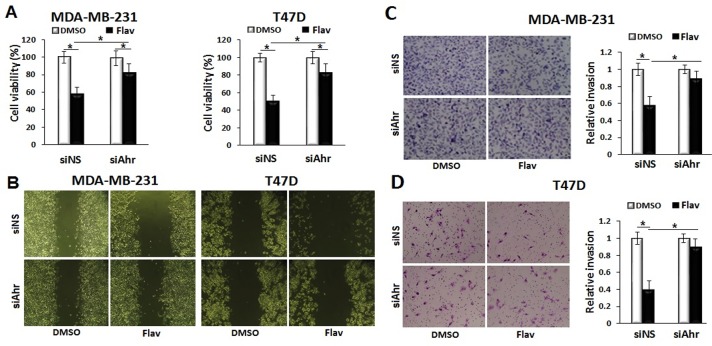
The inhibitory effects of Flavipin on breast cancer cells are Ahr-dependent. (A) The inhibition of proliferation of MDA-MB-231 cells with Flavipin (200 μmol/L) was mitigated with siAhr after 48 h treatment. The cells were transfected with siAhr or siNS by electroporation. Cell proliferation was quantified calorimetrically at 540 nm using CCK-8 kit. (B) Depletion of Ahr by siAhr abrogated the inhibitory effects of Flavipin on migration of breast cancer cells. siAhr mitigated the inhibitory effects of Flavipin on invasion of (C) MDA-MB-231 and (D) T47D cells. Invasiveness of cancer cells were studied using Boyden chamber. The invaded cells were stained with Giemsa and counted in 4 different microscopic fields. Data are shown as mean ± SD from representative experiment studied in triplicates. *P<0.05.

To examine if the inhibitory effects of Flavipin on breast cancer cells were Ahr-dependent, we transfected cells with siAhr or siNS before Flavipin treatment. The data presented in Fig 4A show that knockdown of Ahr attenuated the suppressive effect of Flavipin on proliferation of MDA-MB-231 and T47D cells. Further confirming the role of Ahr on the inhibitory activities of Flavipin on breast cancer cells, depletion of Ahr significantly reversed the suppressive effects of Flavipin on migration (Fig 4B) and invasion of MDA-MB-231 and T47D cells (Fig 4C and 4D). Together, these results suggested that the inhibitory activities of Flavipin on both breast cancer cell lines were mediated mainly by Ahr.

### Flavipin induced Sox4-targeting miR-212/132 cluster

**Fig 5:**
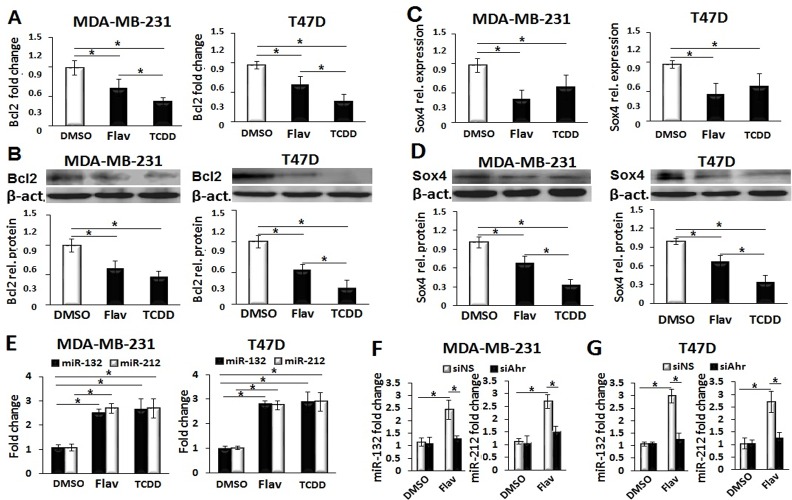
Flavipin suppresses Bcl2 and induces Sox4-targeting miR-212/132. Flavipin (200 μmol/L) or TCDD (10 nmol/L) exerted comparable effects by suppressing mRNA (A) and protein (B) levels of Bcl2 in MDA-MB-231 and T47D cells in 48 h. Quantification was performed by real-time PCR and western blot. (C, D) Gene expression of Sox4 was reduced with Flavipin or TCDD treatment. (E) Flavipin and TCDD induced miR-212/132 expression in both cell lines. (F, G) The Flavipin-mediated expression of miR-212/132 was Ahr-dependent. The cells were transfected with siAhr or siNS by electroporation. Data are shown as mean ± SD from representative experiment studied in triplicates. *P<0.05.

To provide a mechanistic explanation of the inhibitory activities of Flavipin on breast cancer cells, we examined selected anti-apoptotic (Bcl2), adhesion (ITGA4) and pro-metastatic (Sox4) markers. As shown in Fig 5A–5D and S4 Fig, Flavipin exerted suppressive effects on the mRNA and protein levels of Bcl2, ITGA4 and Sox4. Recently, we found that TCDD suppresses invasion of breast cancer cells by inducing the Sox4-targeting miR-212/132 cluster. Therefore, we examined if Flavipin exerted similar effects. Interestingly, Flavipin upregulated the expression of miR-212/132 cluster to a comparable level with that obtained with TCDD treatment (Fig 5E). Next, siAhr was used to examine if the enhancing effects of Flavipin on the expression of miRNA cluster were Ahr-mediated. Inhibition of Ahr clearly indicated that Flavipin induced miR-212/132 cluster in an Ahr-dependent fashion (Fig 5F and 5G). To further investigate the role of Sox4 in the inhibitory effects of Flavipin on migration and invasion, Sox4 was overexpressed in Flavipin-treated breast cancer cells. Over-expression of Sox4 obviously mitigated the inhibitory effects of Flavipin on migration and invasion of MDA-MB-231 and T47D cells (S5 and S6 Figs).

## Discussion

The Ahr is a unique environmentally responsive transcription factor activated by a wide range of exogenous aromatic hydrocarbons. Extensive evidences have clearly indicated the modulatory role of the ligand-activated Ahr in different types of cancers [26–28]. In breast cancer, previous studies have demonstrated that the agonist-activated Ahr modulates invasiveness and metastasis of breast cancer cells, the main causes for tumor recurrence, suggesting Ahr as a promising therapeutic strategy [29, 17, 14, 16]. However, exogenous agonists such as TCDD, the most potent Ahr ligand, which shows inhibitory effects on migration, invasion and metastasis of breast cancer cells is a strong toxin. Therefore, identifying new Ahr agonists from natural origin may open an avenue for safe control over breast cancer metastasis.

Previous studies have identified new Ahr ligands that exhibit biological activities using an *in silico* and *in vitro* means [30, 31]. Applying a computational and *in vitro* approach, we identified Flavipin as a novel potent Ahr agonist. Docking analysis of the interaction between Flavipin and Ahr PAS-A revealed formation of eight hydrogen bonds and binding of Phe115, Leu116 and Ala119 residues of Ahr molecule. These hydrophobic amino acid residues are critical for the Ahr-ligand interaction due to their vital role at the Ahr PAS-A domain and dimerization process [32]. The predicted interaction between Flavipin and PAS-B revealed formation of six hydrogen bonds and binding at Gln83, Asn85, Thr101 and Arg103. Both PAS-A and PAS-B are involved in the heterodimerization process of Ahr with Arnt, and PAS-B is the Ahr domain that interacts with ligands including TCDD [33]. It has been shown that the phenolic and aldehyde components of Ahr ligands interact with Ahr to exert their biological activities [34, 35, 14]. Therefore, such components of Flavipin molecule might have contributed to the Ahr-dependent biological effects on breast cancer cells.

Unlike most of other flavonoids, few studies have investigated the biological activities of Flavipin showing its antibacterial and antifungal properties [22, 36]. In the current study, we found that activation of Ahr by Flavipin suppressed cell proliferation and adhesion in MDA-MB-231 and T47D cells. Consistently, previous reports showed that certain exogenous agonists of Ahr exhibit comparable effects in breast cancer cell lines [16, 36]. The Bcl2 is an anti-apoptotic protein that controls the release of cytochrome from mitochondria [37]. Mechanistically, our results showed that Flavipin suppressed Bcl2 expression, which might have contributed to the anti-proliferative effect of Flavipin. The ITGA4, also known as CD49D, is an integrin α4 chain subunit that heterodimerizes with different β chain proteins to work as surface adhesion receptors that mediate cell adhesion to the extra cellular matrix [38]. Therefore, we speculated that the downregulation of ITGA4 contributed, at least partially, to the suppressive effects of Flavipin on adhesion of MDA-MB-231 and T47D cells.

The findings of the current study indicated that Flavipin exerts inhibitory effects on migration of the MDA-MB-231 and T47D cells. In lines, the suppressive effects of certain Ahr ligands on migration of breast cancer cells are documented [17, 14]. The migration and adhesion are interlinked in cancer prognosis [39]. The cell migration process involves binding of fibronectin to the integrins [40], which is critical to cancer cell invasiveness [40]. These adhesion receptors include the integrins α3β1, α4β1, α5β1, α8β1, αvβ1, αvβ3, and αvβ6 [41]. Among them, α4β1 is the classical fibronectin-binding integrin due to its ligand specificity [42]. Blocking the function of this integrin suppresses the invasiveness capacity of different cancer cells *in vitro* and *in vivo* [42, 43]. In addition, the proliferative characters of breast cancer cells trigger adhesion and migration [36, 44]. Therefore, the suppressive effects of Flavipin on Bcl2 and ITGA4 in the examined breast cancer cells could be among the reasons that contributed to the inhibited migration.

Recent studies have shown that ligand-activated Ahr suppresses invasiveness of different cancer types [45, 14]. In this study, we show consistent results using Flavipin as a novel Ahr agonist. The miRNAs are small non-coding RNAs that are emerging as a new avenue to provide mechanistic explanations for different modulatory effects of Ahr [9, 46, 47]. In this regard, our previous report provided a miR-based mechanistic explanation for the inhibitory role of natural and synthetic Ahr agonists on invasion of MDA-MB-231 and T47D cells [16]. This mechanism suggests that the agonist-activated Ahr induces miR-212/132 cluster that synergistically targets the pro-metastatic factor Sox4 [16]. Consistently, Flavipin induced Sox4-targeting miR-212/132 in an Ahr-dependent fashion. Overexpression of Sox-4 in Flavipin-treated cells mitigated the inhibitory effects of Flavipin on breast cancer migration and invasion. Therefore, Sox4 most likely contributed to the suppressive effects on Flavipin on motility of the breast cancer cells. Furthermore, downregulation of ITGA4 might have partially contributed to the suppressive effect of Flavipin on cell motility. Interestingly, Flavipin exhibited comparable inhibitory effects on both ER-negative and ER-positive cell lines, suggesting an E2-independent mode of action.

Some studies have suggested correlation between endogenous ligand-activated Ahr and breast cancer progression including growth and cell motility and several mechanisms have been proposed [48, 49]. It is important to mention here that the functions of Ahr may differ when ligated with exogenous or endogenous. For example, exogenous ligands of Ahr inhibit BRCA1 oncogene to suppress cancer, whereas, the endogenous ligands exhibit opposing effects [50].

In conclusion, current study introduced Flavipin as a novel Ahr agonist, and provided evidences on its inhibitory activities on growth and motility of breast cancer cells. In addition, it provided mechanistic elucidation for the reported antitumor properties, suggesting Flavipin as a fascinating candidate to control metastasis in breast cancer patients.

## Supporting Information

S1 Fig The *in silico* docking of Lonchophorcol A binding potential to human Ahr. Lonchophorcol A binds to Ahr at Thr264 and Ala119. The yellow-dotted lines indicates the hydrogen bonds.
(JPG)

S2 Fig The *in silico* molecular modeling of human Ahr PAS-B. (A) Superimposition of predicted 3D structure of Ahr PAS-B with the Arnt PAS-B. (B) The Ramachandran plot of predicted Ahr PAS-B and Arnt PAS-B.
(TIF)

S3 Fig Flavipin suppresses adhesion of breast cancer cells. The T47D and MDA-MB-231 cells were seeded in 6-well plate and incubated overnight in complete medium to attach, then washed with PBS to remove the non-adherent cells. Cells were then treated with DMSO or Flavipin (200 μmol/L) in the charcoal-stripped medium for 48 h, and washed two times with PBS before microscopic examination. The T47D medium contained 10 nmol/L E2.
(TIF)

S4 Fig Flavipin suppresses ITGA4 in breast cancer cells. The T47D and MDA-MB-231 cells were seeded in 10 cm plate and incubated overnight in complete medium to attach, then treated with DMSO or Flavipin (200 μmol/L) in the charcoal-stripped medium for 48 h. The T47D medium contained 10 nmol/L E2. (A) ITGA4 mRNA. The mRNA was quantified by real-time PCR using specific probe. (B) ITGA4 protein. The cells were then lysed and ITGA4 protein was quantified using western blotting method using specific antibodies. Band intensities were quantified using ImageJ software. Data are shown as mean ± SD from representative experiment studied in triplicates. *P<0.05.
(TIF)

S5 Fig Sox4 mitigates the inhibitory effects of Flavipin on migration of breast cancer cells. Sox4-expressing or empty vector and incubated for 6 h to recover. The cell were seeded in 6-well plate in a complete medium and allowed to attach overnight, then treated with DMSO or Flavipin (200 μmol/L) in the charcoal-stripped medium for 24 h. A line was made at the central axis of the wells. The T47D medium was supplemented with 10 nmol/L E2.
(TIF)

S6 Fig Sox4 mitigates the inhibitory effects of Flavipin on invasion of breast cancer cells. The cells were transfected by electroporation with Sox4-expressing or empty vector and incubated for 6 h to recover. Cell invasion was studied using Boyden chamber. The cells were suspended in serum-free medium and placed in the trans-well, the lower well contained complete medium. Invaded cells were counted in four microscopic field. Data are shown as mean ± SD from representative experiment studied in triplicates. *P<0.05.
(TIF)

S1 Table Molecular docking of Ahr agonist candidates. (DOCX)

S2 Table Primary structural properties of human Ahr PAS-B domain. (DOCX)

S3 Table Amino acid composition of human Ahr PAS-B domain. (DOCX)

S4 Table Procheck statistics of 3D structure human Ahr PAS-B domain. (DOCX)
