# Supplementary figures and images for: Novel Aryl Hydrocarbon Receptor Agonist Suppresses Migration and Invasion of Breast Cancer Cells
Source: PLoS One. 2016 Dec 1;11(12):e0167650. doi: 10.1371/journal.pone.0167650 (PMC5132326; doi:10.1371/journal.pone.0167650)

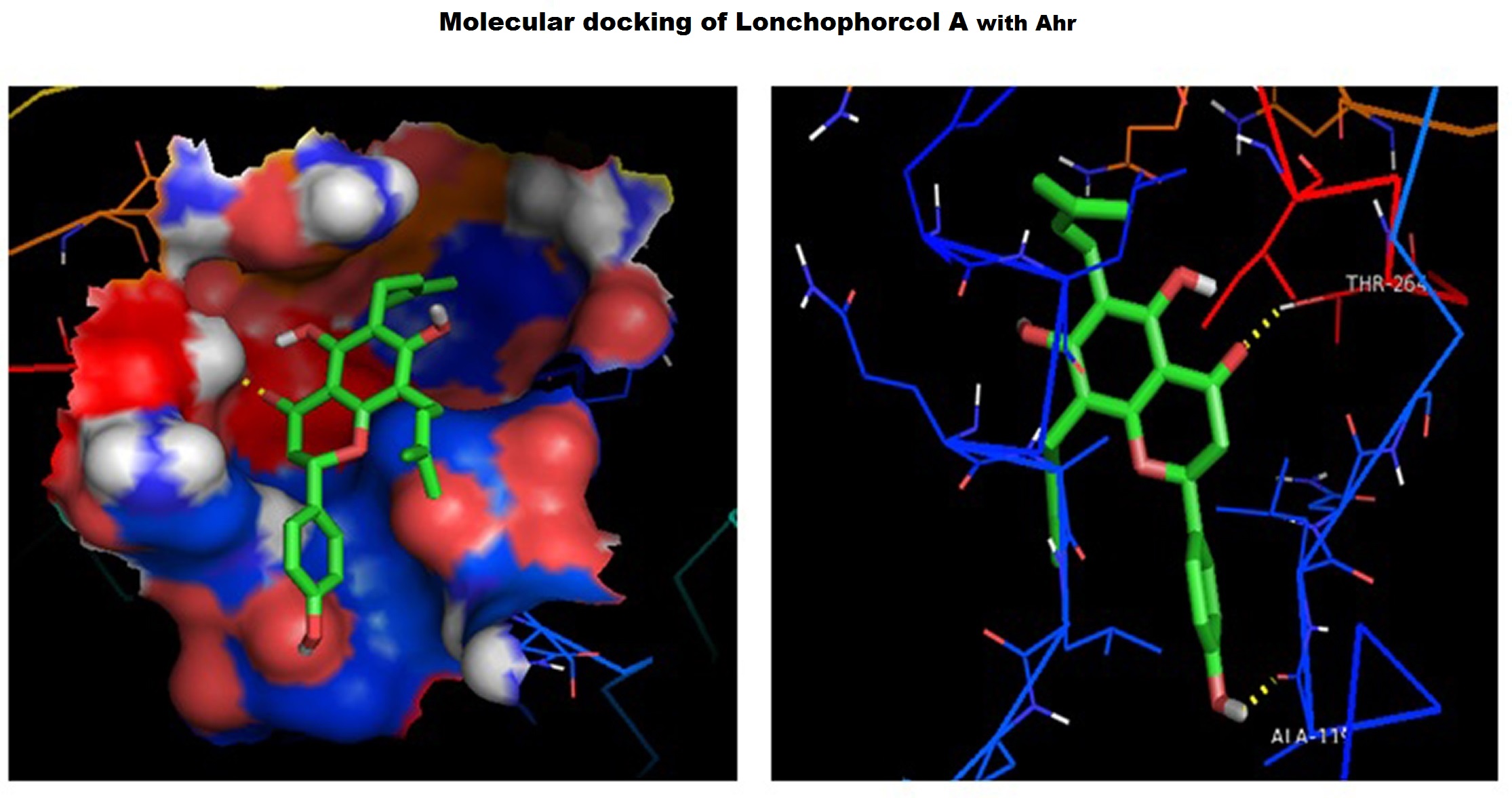

Supplement: S1 Fig — Lonchophorcol A binds to Ahr at Thr264 and Ala119. The yellow-dotted lines indicates the hydrogen bonds. (JPG) [file pone.0167650.s001.jpg]

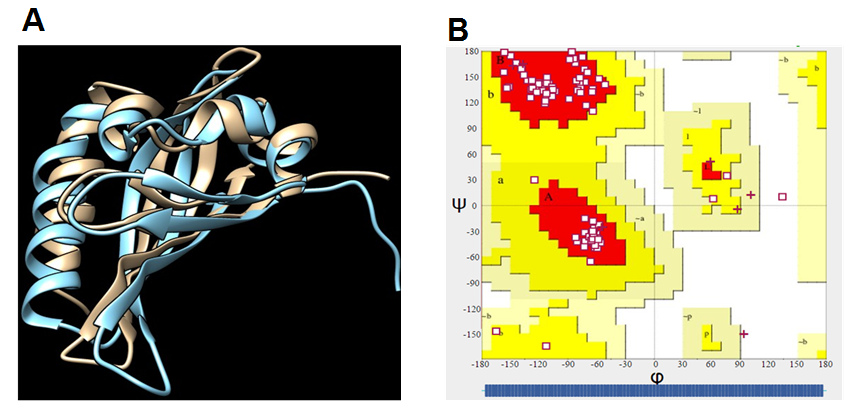

Supplement: S2 Fig — (A) Superimposition of predicted 3D structure of Ahr PAS-B with the Arnt PAS-B. (B) The Ramachandran plot of predicted Ahr PAS-B and Arnt PAS-B. (TIF) [file pone.0167650.s002.tif]

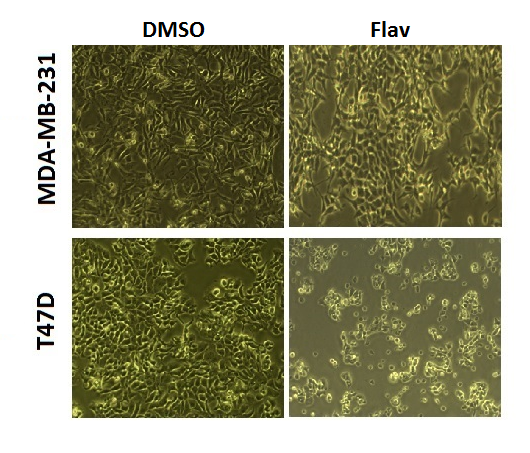

Supplement: S3 Fig — The T47D and MDA-MB-231 cells were seeded in 6-well plate and incubated overnight in complete medium to attach, then washed with PBS to remove the non-adherent cells. Cells were then treated with DMSO or Flavipin (200 μmol/L) in the charcoal-stripped medium for 48 h, and washed two times with PBS before microscopic examination. The T47D medium contained 10 nmol/L E2. (TIF) [file pone.0167650.s003.tif]

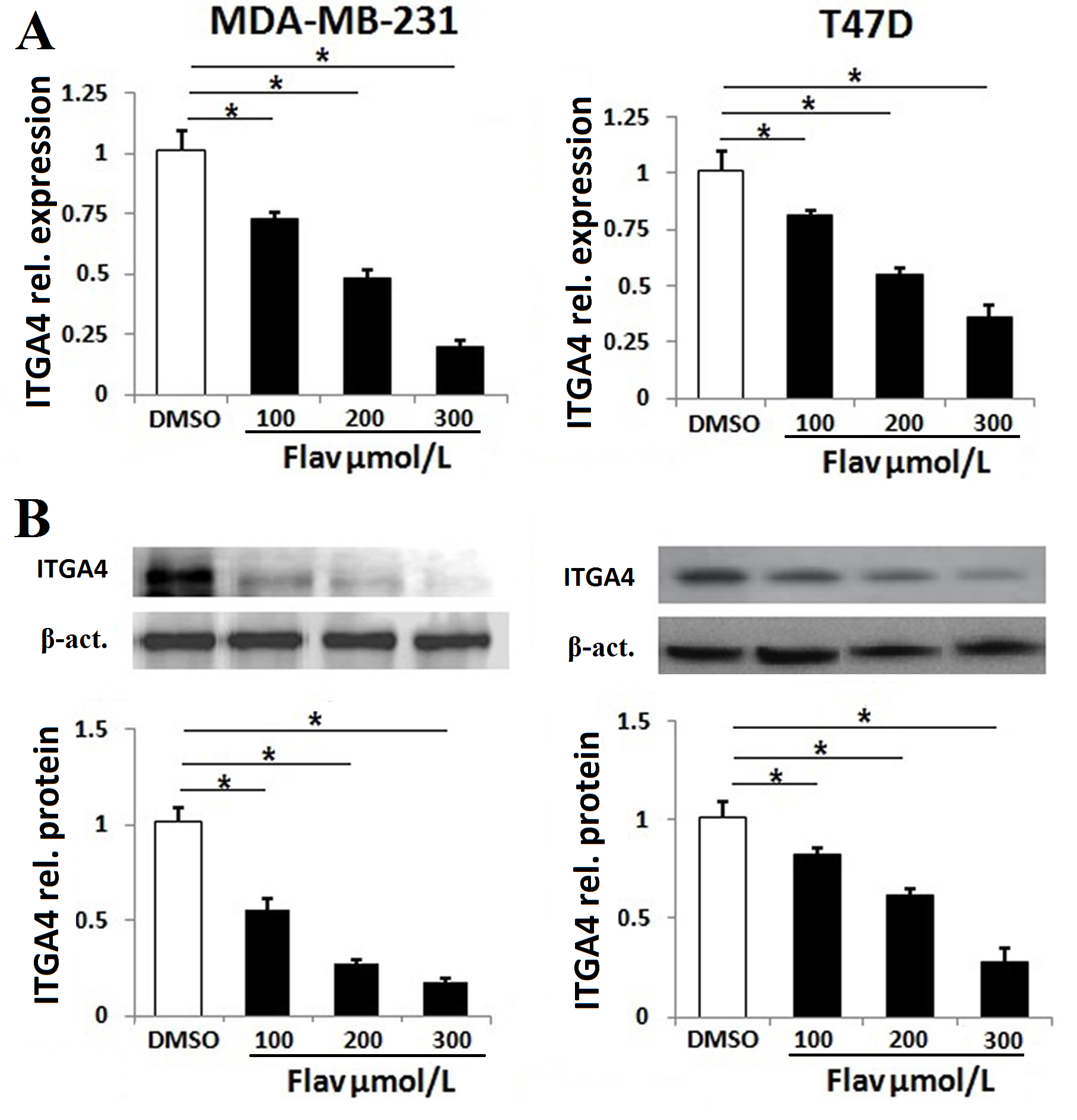

Supplement: S4 Fig — The T47D and MDA-MB-231 cells were seeded in 10 cm plate and incubated overnight in complete medium to attach, then treated with DMSO or Flavipin (200 μmol/L) in the charcoal-stripped medium for 48 h. The T47D medium contained 10 nmol/L E2. (A) ITGA4 mRNA. The mRNA was quantified by real-time PCR using specific probe. (B) ITGA4 protein. The cells were then lysed and ITGA4 protein was quantified using western blotting method using specific antibodies. Band intensities were quantified using ImageJ software. Data are shown as mean ± SD from representative experiment studied in triplicates. *P<0.05. (TIF) [file pone.0167650.s004.tif]

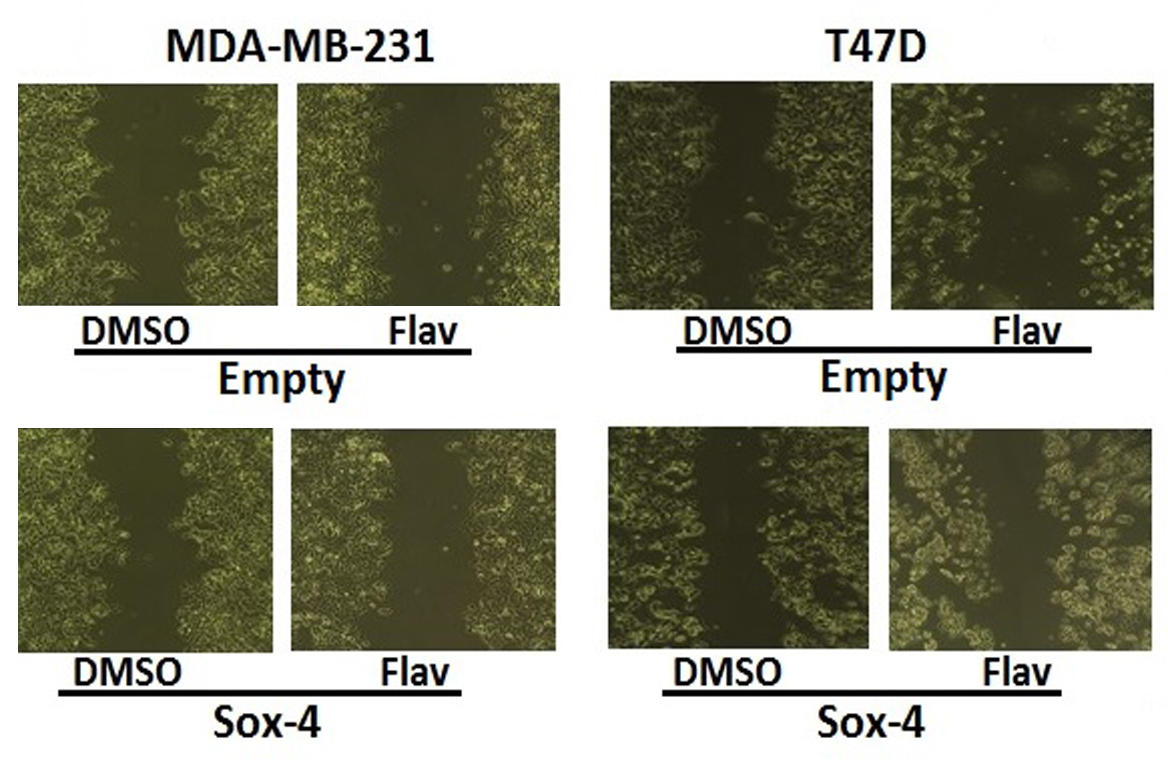

Supplement: S5 Fig — Sox4-expressing or empty vector and incubated for 6 h to recover. The cell were seeded in 6-well plate in a complete medium and allowed to attach overnight, then treated with DMSO or Flavipin (200 μmol/L) in the charcoal-stripped medium for 24 h. A line was made at the central axis of the wells. The T47D medium was supplemented with 10 nmol/L E2. (TIF) [file pone.0167650.s005.tif]

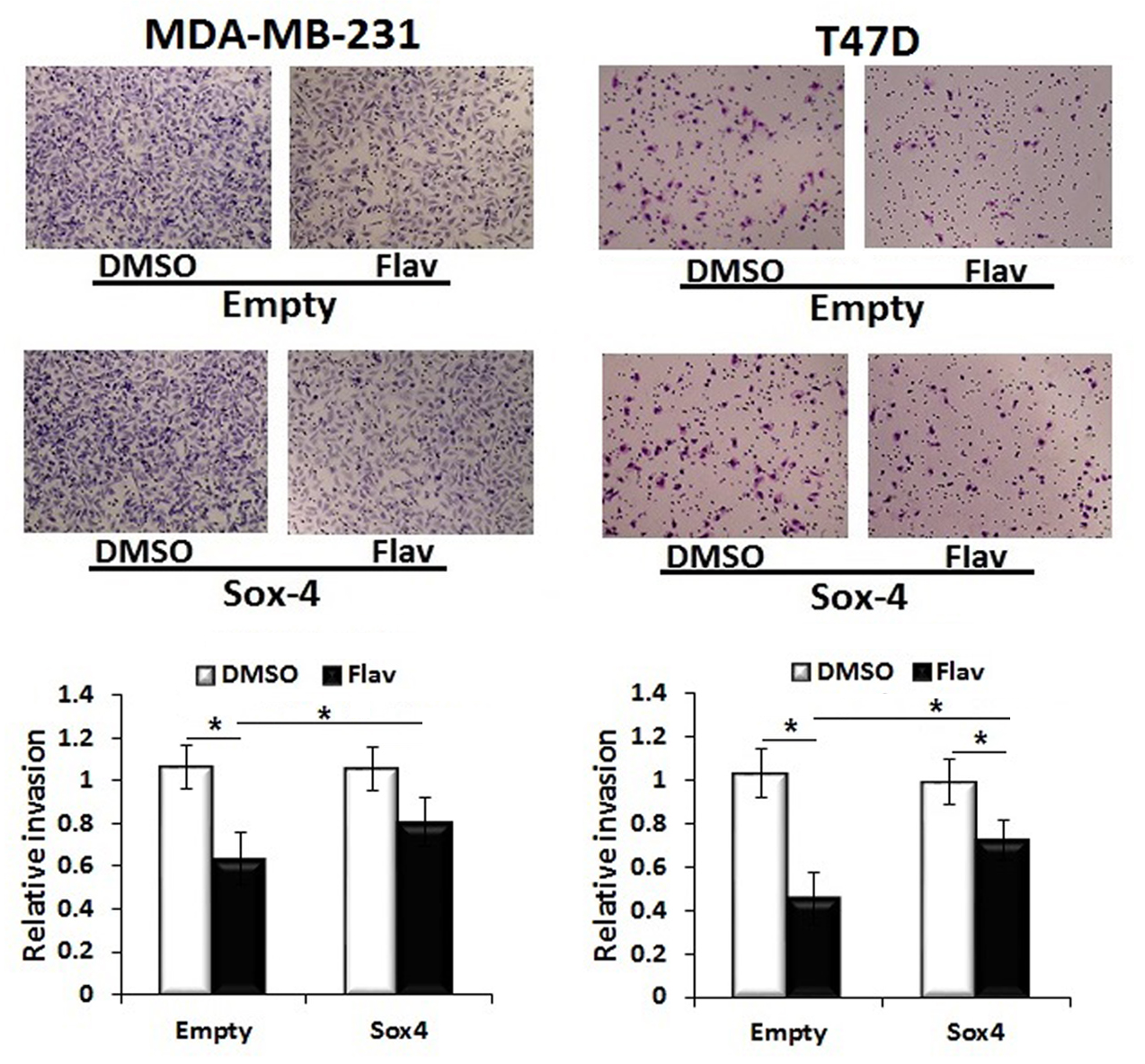

Supplement: S6 Fig — The cells were transfected by electroporation with Sox4-expressing or empty vector and incubated for 6 h to recover. Cell invasion was studied using Boyden chamber. The cells were suspended in serum-free medium and placed in the trans-well, the lower well contained complete medium. Invaded cells were counted in four microscopic field. Data are shown as mean ± SD from representative experiment studied in triplicates. *P<0.05. (TIF) [file pone.0167650.s006.tif]
