# Supplementary material for: Novel Aryl Hydrocarbon Receptor Agonist Suppresses Migration and Invasion of Breast Cancer Cells
Source: PLoS One. 2016 Dec 1;11(12):e0167650. doi: 10.1371/journal.pone.0167650 (PMC5132326; doi:10.1371/journal.pone.0167650)
Supplement: S1 Table — (DOCX) [file pone.0167650.s007.docx]

**S1 Table**

| **Ligand** | **Receptor** | **Binding**  **energy** | **Ligand**  **efficiency** | **Intermole**  **energy** | **Ligand atoms (ring)** | **Docked amino**  **acid residue (bond length)** |
| --- | --- | --- | --- | --- | --- | --- |
| Lonchocarpol A  (Pubchem id: 124035) | Ahr  PAS-A | -4.35 | -0.18 | -6.28 | - C-11’O - C-25’OH | - Chain A THR`264/HG1   (2.4 Å)   - Chain A ALA`119/O   (2.1 Å) |
| Epicorazines B  (Pubchem id: 73891006) | Ahr  PAS-A | No interaction |  |  |  |  |
| Epicorazines A  (Pubchem id: 57383998) | Ahr  PAS-A | No interaction |  |  |  |  |
