# Supplementary material for: Novel Aryl Hydrocarbon Receptor Agonist Suppresses Migration and Invasion of Breast Cancer Cells
Source: PLoS One. 2016 Dec 1;11(12):e0167650. doi: 10.1371/journal.pone.0167650 (PMC5132326; doi:10.1371/journal.pone.0167650)
Supplement: S2 Table — (DOCX) [file pone.0167650.s008.docx]

S2 Table

| **Parameters** | **Theoretical Prediction** |
| --- | --- |
| Molecular weight (kDa) | 121.57 |
| Isoelectric point | 9.76 |
| Extinction coefficient (M^-1^ cm^-1^ at 280nm) | 18575 |
| Estimated half-life (h):   - Mammalian reticulocytes (in-vitro) - Yeast (in-vivo) - *Escherichia coli* (in-vivo) | - 7.2 - >20 - >10 |
| Instability index | 20.63 |
| Aliphatic index | 83.62 |
| Grand average of hydropathicity (GRAVY) | -0.283 |
