# Supplementary material for: Novel Aryl Hydrocarbon Receptor Agonist Suppresses Migration and Invasion of Breast Cancer Cells
Source: PLoS One. 2016 Dec 1;11(12):e0167650. doi: 10.1371/journal.pone.0167650 (PMC5132326; doi:10.1371/journal.pone.0167650)
Supplement: S3 Table — (DOCX) [file pone.0167650.s009.docx]

S3 Table

| **Amino acid** | **Composition** | **Percentage %** |
| --- | --- | --- |
| Ala (A) | 6 | 5.7% |
| Arg (R) | 9 | 8.6% |
| Asn (N) | 5 | 4.8% |
| Asp (D) | 4 | 3.8% |
| Cys (C) | 3 | 2.9% |
| Gln (Q) | 3 | 2.9% |
| Glu (E) | 4 | 3.8% |
| Gly (G) | 8 | 7.6% |
| His (H) | 3 | 2.9% |
| Ile (I) | 9 | 8.6% |
| Leu (L) | 9 | 8.6% |
| Lys (K) | 7 | 6.7% |
| Met (M) | 3 | 2.9% |
| Phe (F) | 5 | 4.8% |
| Pro (P) | 3 | 2.9% |
| Ser (S) | 4 | 3.8% |
| Thr (T) | 9 | 8.6% |
| Trp (W) | 2 | 1.9% |
| Tyr (Y) | 5 | 4.8% |
| Val (V) | 4 | 3.8% |
