# Supplementary material for: Novel Aryl Hydrocarbon Receptor Agonist Suppresses Migration and Invasion of Breast Cancer Cells
Source: PLoS One. 2016 Dec 1;11(12):e0167650. doi: 10.1371/journal.pone.0167650 (PMC5132326; doi:10.1371/journal.pone.0167650)
Supplement: S4 Table — (DOCX) [file pone.0167650.s010.docx]

S4 Table

| 1. **Ramachandran Plot statistics** | | |
| --- | --- | --- |
| **Ramachandran Plot statistics** | **No. of residues** | **Percentage** |
| Most favoured regions [A,B,L] | 85 | (92.40 %) |
| Additionally allowed regions [a,b,l,p] | 6 | (6.50 %) |
| Generously allowed regions [_˜_a, _˜_b, _˜_l, _˜_p] | 0 | (0%) |
| Disallowed regions [XX] | 1 | (1.10 %) |
| Total non-glycine and non-proline residues | 92 | (100 %) |
| End-residues (excl. Gly and proline) | 2 |  |
| Glycine residues | 8 |  |
| Proline residues | 3 |  |
| Total number of residues | 105 |  |
| 1. **G-Factors parameters** | | |
| **G-factor parameters** | **Score** | **Average score** |
| Dihedral angles:   - Phi-psi distribution - Chi1-chi2 distribution - Chi1 only - Chi3 & chi4 - Omega | - -0.07 - -0.22 - 0.29 - 0.58 - -0.16 | -0.02 |
| Main chain covalent forces:   - Main chain bond length - Main chain bond angles | - 0.59 - -0.59 | -0.09 |
| Overall average: |  | -0.01 |
